# Interindividual variation in gene expression responses and metabolite formation in acetaminophen-exposed primary human hepatocytes

**DOI:** 10.1007/s00204-015-1545-2

**Published:** 2015-06-24

**Authors:** Marlon J. A. Jetten, Ainhoa Ruiz-Aracama, Maarten L. J. Coonen, Sandra M. Claessen, Marcel H. M. van Herwijnen, Arjen Lommen, Joost H. M. van Delft, Ad A. C. M. Peijnenburg, Jos C. S. Kleinjans

**Affiliations:** Department of Toxicogenomics, Maastricht University, Universiteitssingel 50, Room 4.112 UNS 50, 6229 ER Maastricht, The Netherlands; RIKILT, Institute of Food Safety, Wageningen UR, PO Box 230, 6700 AE Wageningen, The Netherlands

**Keywords:** Interindividual variation, Primary human hepatocytes, DNA methylation, Gene expression, Aflatoxin b1, Benzo(a)pyrene

## Abstract

Acetaminophen (APAP) is a readily available over-the-counter drug and is one of the most commonly used analgesics/antipyretics worldwide. Large interindividual variation in susceptibility toward APAP-induced liver failure has been reported. However, the exact underlying factors causing this variability in susceptibility are still largely unknown. The aim of this study was to better understand this variability in response to APAP by evaluating interindividual differences in gene expression changes and APAP metabolite formation in primary human hepatocytes (PHH) from several donors (*n* = 5) exposed in vitro to a non-toxic to toxic APAP dose range. To evaluate interindividual variation, gene expression data/levels of metabolites were plotted against APAP dose/donor. The correlation in APAP dose response between donors was calculated by comparing data points from one donor to the data points of all other donors using a Pearson-based correlation analysis. From that, a correlation score/donor for each gene/metabolite was defined, representing the similarity of the omics response to APAP in PHH of a particular donor to all other donors. The top 1 % highest variable genes were selected for further evaluation using gene set overrepresentation analysis. The biological processes in which the genes with high interindividual variation in expression were involved include liver regeneration, inflammatory responses, mitochondrial stress responses, hepatocarcinogenesis, cell cycle, and drug efficacy. Additionally, the interindividual variation in the expression of these genes could be associated with the variability in expression levels of hydroxyl/methoxy-APAP and C8H13O5N-APAP-glucuronide. The before-mentioned metabolites or their derivatives have also been reported in blood of humans exposed to therapeutic APAP doses. Possibly these findings can contribute to elucidating the causative factors of interindividual susceptibility toward APAP.

**Electronic supplementary material:**

The online version of this article (doi:10.1007/s00204-015-1545-2) contains supplementary material, which is available to authorized users.

## Introduction

Acetaminophen (APAP) is one of the most commonly used analgesics/antipyretics worldwide. Thereby, it is a readily available over-the-counter drug. Apart from its beneficial effects as a pharmaceutical, APAP is also the most common cause of acute liver toxicity in Europe and the USA (Lee 2007). Cytochrome P450 enzymes in the liver metabolize APAP to the oxidative metabolite *N*-acetyl-*p*-benzoquinone imine (NAPQI), which is thought to cause the toxic effects of APAP through protein adduct formation, leading to oxidative stress and finally liver damage (Dahlin et al. 1984a). The molecular mechanism behind further progression of APAP toxicity still is not fully elucidated; the involvement of multiple mechanisms of toxicity, like inflammatory responses and oxidative stress, has been suggested (Jaeschke et al. 2012).

The main body of our knowledge on the toxicological, or more specific hepatotoxic, mechanisms of compounds, including APAP, is based on data collected from studies using animal models (Hinson et al. 2010). However, increasing criticism on the usability and applicability of animal data to the human situation has developed over the last years (Greek and Menache 2013). Therefore, there is a growing need for human in vitro models such as hepatic cell lines, liver slices, or primary hepatocytes cultures, to facilitate human-based research.

Large interindividual differences in response to xenobiotic exposure between humans have been documented (Court et al. 2001). In the domains of toxicology/pharmacology, many attempts are being undertaken to gain a better understanding of the factors that are causative of this interindividual variation. Environmental factors as well as genetic factors have been proposed to contribute to the variation in drug responses between human individuals. Over the last decade, with the rise of the whole genome omics techniques, it has become feasible to perform more complete/in-depth analysis of the genomic components contributing to interindividual variation in the human population (Berg 2014).

Also, the metabolism of APAP is known to show large interindividual variability (Court et al. 2001). Genetic factors including many biotransformation-related genes, such as UDP-glucuronosyltransferases and cytochrome P450 enzymes, have been suggested to be causative of the variation in APAP-induced adverse effects observed between individuals (Court et al. 2001; Fisher et al. 2000; Polasek et al. 2006; Yasar et al. 2013). While some individuals seem to be able to endure APAP doses considerably exceeding the recommended maximal daily dose, others are at risk of liver toxicity due to APAP much closer to the recommended dose window (Sabate et al. 2011). The relatively high frequency of unintentional overdosing has recently led the FDA to adjusting recommendations on safe APAP dosage use by lowering the maximal therapeutic daily dose and decreasing the single dose units of APAP (Turkoski 2010).

However, not only interindividual differences do occur at supra-therapeutic doses, but also (sub-) therapeutic doses of APAP have been shown to cause interindividual variation in APAP metabolite levels, as well as in mRNA and miRNA expression levels (Jetten et al. 2012). In the same study, regulation of biological processes known to be related to the liver toxicity response after APAP overdosing could be detected at lower and supposedly non-toxic doses.

In this study, we aim at investigating the interindividual differences in response to a non-toxic to toxic APAP dose range, using an in vitro cell model consisting of primary human hepatocytes of several donors. The interindividual differences in APAP metabolite formation and gene expression responses were considered and compared in an attempt to pinpoint the factors that could be causative of interindividual variation in APAP metabolism.

## Materials and methods

### Cell culture and treatment

#### Primary human hepatocytes

Cryopreserved PHH of five individuals (see Supplementary Table 1 for donor demographics) were purchased from Life Technologies (Gibco). Cells were cultured in 12-well plates in a collagen sandwich, according to the supplier’s protocol (Invitrogen 2009). The following culture media were used: cryopreserved hepatocyte recovery medium (CHRM, Gibco) for thawing, William’s medium E (WME) + Glutamax (Gibco) substituted with 10 % FCS (Gibco), 0.02 % penicillin/streptomycin (Gibco), and 0.1 U/ml insulin (Invitrogen) for seeding/attaching and WME + Glutamax substituted with 0.02 % Pen/Strep, 0.1 U/ml insulin, and 0.02 mg/ml hydrocortisone (Sigma-Aldrich) for culturing/exposure. After thawing, viability of the cells was checked by a trypan blue (CAS no. 72-57-1, Sigma-Aldrich) exclusion test as instructed in the supplier’s protocol (Invitrogen 2009). All viability scores were in accordance with those listed by the supplier. Hepatocytes were exposed for 24 h to 0, 0.2, 2, or 10 mM APAP (CAS no. 103-90-2, Sigma-Aldrich) dissolved in culture medium. The doses, representing no observed effect level (NOEL), lowest observed effect level (LOEL = non-toxic), and toxic dose, respectively, were chosen based on the available literature (Bannwarth et al. 2001; Borin and Ayres 1989; Critchley et al. 2005; Douglas et al. 1996; Kamali 1993; Kienhuis et al. 2009; Portolés et al. 2003; Rygnestad et al. 2000; Tan and Graudins 2006; Yin et al. 2001).

### Transcriptomic sample preparation

#### Total RNA isolation

QIAzol (0.5 ml, QIAGEN) was used to isolate total RNA from all samples according to the manufacturer’s protocol. RNA purification was performed with the miRNeasy Mini Kit (Qiagen) as instructed by the manufacturer. Next, the integrity of the RNA was checked with the Bioanalyzer 2100 (Agilent).

#### cDNA preparation/hybridization

From an input of 250 ng RNA, cDNA targets were prepared using the Affymetrix protocol. The procedures as recommended by the manufacturer were applied to hybridize the samples to Affymetrix GeneChip Human Genome U133A plus 2 GeneChip arrays. GeneChips were washed and stained after hybridization with a fluidics station (Affymetrix) and scanned with a GeneArray scanner (Affymetrix). The samples from donor 1 exposed to 10 mM APAP did not pass quality control and were therefore excluded from further analyses.

### Transcriptomic data analysis

The CEL files retrieved in the previous step were subjected to an overall quality control, using arrayanalysis.org, and all arrays were of high quality (Eijssen et al. 2013). Subsequently, data were RMA-normalized and re-annotated using BrainArray’s EntrezGene customCDF_V15.1 (Dai et al. 2005; Lim et al. 2012). Probes with low signal-to-noise ratio (average expression <6) were excluded from further analyses as a data-cleanup step.

### Metabolomic sample preparation

Culture medium from the cells was collected after 24 h and stored at −80 °C until extraction. To extract the metabolites, 6 ml of ice-cold acetone was added to 1.5 ml of medium in a 10-ml glass tube. The solution was vortexed for 30 s, kept on ice for 12 min, and then centrifuged for 15 min at 2800 rpm at 4 °C. The supernatant was transferred into a 10-ml glass tube and dried under nitrogen. To concentrate the semi-polar metabolites contained in the medium, SPE C18 columns (C18, 500 mg, 3 ml, Bond-Elut, VARIAN) were used. The SPE C18 column was conditioned by running it with methanol including 0.5 % of formic acid (1 ml twice) and MilliQ including 0.5 % of formic acid (1 ml twice). Once the dried pellet was re-suspended in 1 ml of MilliQ (with 0.5 % formic acid), it was applied to the column. The column was then washed twice with 1 ml of MilliQ, after which the components of interest were eluted with 1 ml of methanol and dried under nitrogen. For U-HPLC-Orbitrap analysis, the dried polar fraction was re-suspended in 400 μl of MilliQ with 0.1 % formic acid.

#### U-HPLC-Orbitrap MS analysis

Experimental setups and procedures as described before have been used with some slight modifications as defined below (Lommen 2009; Lommen et al. 2011; Ruiz-Aracama et al. 2011). The gradient was similar to the one used in Jetten et al. (2012) with small modifications. The initial eluent composition, 100 % A, was changed to 85% A and 15 % B in 15 min. Afterward, the composition of B was increased to 30 % in 10 min and subsequently increased in 3 min to 90 %, remaining at this composition for 5 min prior to the next injection. A capillary temperature of 250 °C with a sheath and auxiliary gas flow of 19 and 7 arbitrary units were used, respectively.

### Metabolomic data analysis

Visual inspection of the three technical replicates of each sample showed a high degree of reproducibility. All MS data were preprocessed and aligned using the in-house developed program, metAlign (Lommen 2009). A targeted search for the metabolites of APAP previously described in Jetten et al. (2012) was executed. For targeted analyses, Search LCMS, an add-on tool for metAlign, was used (Lommen 2009). Briefly, a list of masses of interest was composed based on our previous in vivo study with human volunteers (Jetten et al. 2012) and some further data available from mice (Chen et al. 2008a). This list was loaded into Search LCMS, which returned the amplitudes of the masses of interest.

U-HPLC-Orbitrap MS data were preprocessed as described in previous papers (Lommen 2009; Lommen et al. 2011) to obtain ultra-precise (sub-ppm) mass data (calibration using internal masses and PEG200, PEG300, PEG600 as external masses). Metabolites were considered to be present when retention times were analogous to earlier experiments and average accurate masses were below ±3 ppm; nearly all average masses of metabolites were within 1 ppm. For some metabolites previously not found, including hydroxy-APAP, methoxy-APAP, and 3,3′-biacetaminophen, retention times were related to those derived from the literature if possible (Chen et al. 2008a; Jetten et al. 2012). Metabolite expression levels for methoxy-APAP-glucuronide-1/2 and hydroxy-APAP-glutathione in samples of donor 1 exposed to 10 mM APAP could unfortunately not be determined due to technical issues.

#### Metabolite visualization

To create a metabolic map based on available literature, a pathway visualization tool, PathVisio, was used (Chen et al. 2008b; Daykin et al. 2002; van Iersel et al. 2008). LC–MS data were visualized for each donor and per dose (corrected for control levels, 0 mM APAP). Log-transformation of the data resulted in range with a minimum of 0 and a maximum of 5.

### Data integration and visualization; interindividual variation

Expression data (log2-scaled intensities) of all genes passing the selection as described under ‘transcriptomic data analysis’ and levels of all of the identified metabolites were plotted against APAP dose per donor (*X*-axis: dose 0, 0.2, 2, and 10 mM APAP; *Y*-axis: log-scaled gene expression/metabolite levels; line: donor, *n* = 5) using R 2.15.3 (R Core Team 2013). For clarification purposes, a representative plot is provided in Supplementary Figure 1. To estimate the correlation in APAP dose response between donors, data points from one donor were compared to the data points of all other donors using a Pearson-based correlation analysis, which resulted in the following comparisons: D1–D2, D1–D3, D1–D4, D1–D5, D2–D3, D2–D4, D2–D5, D3–D4, D3–D5, and D4–D5. Then, the absolute correlation coefficients of each donor were summed to generate an arbitrary correlation score per donor for each gene/metabolite (Score D1, Score D2, Score D3, Score D4, and Score D5; see supplementary Figure 1). This ‘score’ now represents the similarity of a particular donor to all other donors in expression response for a particular gene/metabolite following APAP exposure; the donor with the lowest score is most aberrant from all other donors (showed the least correlation with the other donors). In order to select the most variable genes between donors, standard deviations (SD) of the donor scores per gene/metabolite were calculated and ranked. The top 1 % ranked genes (score *SD* > 2.52) were selected for further analysis, since these exhibit the most interdonor variability (Table 1).

**Table 1:** List of the top 1 % most variable genes based on Pearson correlation analysis

EntrezGeneID	Gene name	Functional description according to GeneCards
92	ACVR2A	Kinase receptor
*513*	*ATP5D*	*Subunit of mitochondrial ATP synthase*
595	CCND1	Cyclin family
*617*	*BCS1L*	*Complex III of the mitochondrial respiratory chain*
988	CDC5L	Cell cycle regulator important for G2/M transition
1545	CYP1B1	Cytochrome P450 superfamily
1611	DAP	Mediator of programmed cell death
2669	GEM	GTP-binding proteins, receptor-mediated signal transduction
2766	GMPR	NADPH-dependent reductive deamination of GMP to IMP
3276	PRMT1	Methyltransferase
4302	MLLT6	Myeloid/lymphoid or mixed-lineage leukemia
4615	MYD88	Myeloid differentiation primary response
5201	PFDN1	Member of the prefolding beta subunit family
5287	PIK3C2B	PI3-kinases play roles in signaling pathways involved in cell proliferation, oncogenic transformation, cell survival, cell migration, and intracellular protein trafficking
5300	PIN1	Regulation of cell growth, genotoxic, and other stress responses, the immune response, induction and maintenance of pluripotency, germ cell development, neuronal differentiation, and survival
5523	PPP2R3A	Negative control of cell growth and division
5550	PREP	Maturation and degradation of peptide hormones and neuropeptides
5584	PRKCI	Protective role against apoptotic stimuli, is involved in NF-kappa-B activation, cell survival, differentiation, and polarity and contributes to the regulation of microtubule dynamics in the early secretory pathway
5696	PSMB8	Apoptosis, may be involved in the inflammatory response pathway
5699	PSMB10	Involved in antigen processing to generate class I-binding peptides
5796	PTPRK	Cell growth, differentiation, mitotic cycle, and oncogenic transformation
6612	SUMO3	Posttranslationally modify numerous cellular proteins and affect their metabolism and function, such as nuclear transport, transcriptional regulation, apoptosis, and protein stability
6942	TCF20	Stimulates the activity of various transcriptional activators such as JUN, SP1, PAX6, and ETS1, suggesting a function as a co-activator
7264	TSTA3	Cell–cell interactions, including cell–cell recognition; in cell–matrix interactions; in detoxification processes
7572	ZNF24	Transcription repressor activity
7965	AIMP2	Functions as a pro-apoptotic factor
8270	LAGE3	???
*8310*	*ACOX3*	*Desaturation of 2-methyl branched fatty acids in peroxisomes*
8985	PLOD3	Hydroxylation of lysyl residues in collagen-like peptides
9343	EFTUD2	A component of the spliceosome complex which processes precursor mRNAs to produce mature mRNAs
*9361*	*LONP1*	*Mediates the selective degradation of misfolded, unassembled, or oxidatively damaged polypeptides in the mitochondrial matrix assembly of inner membrane protein complexes and participates in the regulation of mitochondrial gene expression and maintenance of the integrity of the mitochondrial genome*
9470	EIF4E2	EIF4E2 gene promoter protein synthesis and facilitates ribosome binding by inducing the unwinding of the mRNA secondary structures
10093	ARPC4	Regulation of actin polymerization and together with an activating nucleation-promoting factor (NPF) mediates the formation of branched actin networks
10189	THOC4	Molecular chaperone. It is thought to regulate dimerization, DNA binding, and transcriptional activity of basic region-leucine zipper (bZIP) proteins
10313	RTN3	Involved in membrane trafficking in the early secretory pathway
10422	UBAC1	Required for poly-ubiquitination and proteasome-mediated degradation of CDKN1B during G1 phase of the cell cycle
10598	AHSA1	May affect a step in the endoplasmic reticulum to Golgi trafficking
10807	SDCCAG3	May be involved in modulation of TNF response
10899	JTB	Required for normal cytokinesis during mitosis. Plays a role in the regulation of cell proliferation
11068	CYB561D2	Acting as an ubiquitin-conjugating enzyme, involved in the regulation of exit from mitosis, cell cycle, protein, ubiquitin-dependent proteolysis, electron transport
11131	CAPN11	Remodeling of cytoskeletal attachments to the plasma membrane during cell fusion and cell motility, proteolytic modification of molecules in signal transduction pathways, degradation of enzymes controlling progression through the cell cycle, regulation of gene expression, substrate degradation in some apoptotic pathways, and an involvement in long-term potentiation
11142	PKIG	PKA inhibitors; protein kinase A has several functions in the cell, including regulation of glycogen, sugar, and lipid metabolism
11252	PACSIN2	Involved in linking the actin cytoskeleton with vesicle formation by regulating tubulin polymerization
*11332*	*ACOT7*	*Catalyze the hydrolysis of acyl-CoAs to the free fatty acid and coenzyme A (CoASH), providing the potential to regulate intracellular levels of acyl-CoAs, free fatty acids, and CoASH*
23243	ANKRD28	Involved in the recognition of phosphoprotein substrates
23325	KIAA1033	Plays a key role in the fission of tubules that serve as transport intermediates during endosome sorting
23558	WBP2	Involved in mediating protein–protein interactions through the binding of polyproline ligands
26100	WIPI2	Probable early component of the autophagy machinery being involved in formation of preautophagosomal structures and their maturation into mature phagosomes
26505	CNNM3	Probable metal transporter
*26520*	*TIMM9*	*Mediate the import and insertion of hydrophobic membrane proteins into the mitochondrial inner membrane*
27075	TSPAN13	Mediate signal transduction events that play a role in the regulation of cell development, activation, growth, and motility
29105	C16orf80	???
29927	SEC61A1	Plays a crucial role in the insertion of secretory and membrane polypeptides into the ER
50640	PNPLA8	Phospholipases which catalyze the cleavage of fatty acids from membrane phospholipids
51094	ADIPOR1	Regulates fatty acid catabolism and glucose levels
51491	NOP16	Involved in ribosome biogenesis
51504	TRMT112	Participates in both methylation of protein and tRNA species
51523	CXXC5	Required for DNA damage-induced ATM phosphorylation, p53 activation, and cell cycle arrest. Involved in myelopoiesis
*51706*	*CYB5R1*	*Involved in desaturation and elongation of fatty acids, cholesterol biosynthesis, drug metabolism, and, in erythrocyte, methemoglobin reduction*
54187	NANS	Functions in the biosynthetic pathways of sialic acids
54606	DDX56	Implicated in a number of cellular processes involving alteration of RNA secondary structure such as translation initiation, nuclear and mitochondrial splicing, and ribosome and spliceosome assembly. May play a role in later stages of the processing of the preribosomal particles, leading to mature 60S ribosomal subunits. Has intrinsic ATPase activity
54941	RNF125	E3 ubiquitin-protein ligase that acts as a positive regulator of T cell activation
55062	WIPI1	May play a role in autophagy
55111	PLEKHJ1	??? phospholipid binding
55238	SLC38A7	Mediates sodium-dependent transport of amino acids
55315	SLC29A3	Plays a role in cellular uptake of nucleosides, nucleobases, and their related analogs
55647	RAB20	Plays a role in the maturation and acidification of phagosomes that engulf pathogens
55700	MAP7D1	Mitotic spindle protein and member of the MAP7 (microtubule-associated protein 7) family of proteins
55743	CHFR	Functions in the antephase checkpoint by actively delaying passage into mitosis in response to microtubule poisons
55898	UNC45A	Plays a role in cell proliferation and myoblast fusion, binds progesterone receptor and HSP90, and acts as a regulator of the progesterone receptor chaperoning pathway
56005	C19orf10	???
*56267*	*CCBL2*	*Encodes an aminotransferase that transaminates kynurenine to form kynurenic acid*
56910	STARD7	???
57409	MIF4GD	Functions in replication-dependent translation of histone mRNAs
64754	SMYD3	Histone methyltransferase
64787	EPS8L2	Is thought to link growth factor stimulation to actin organization, generating functional redundancy in the pathways that regulate actin cytoskeletal remodeling
*64949*	*MRPS26*	*Mammalian mitochondrial ribosomal proteins are encoded by nuclear genes and help in protein synthesis within the mitochondrion*
66036	MTMR9	Thought to have a role in the control of cell proliferation
79056	PRRG4	??? Calcium ion binding
80227	PAAF1	Involved in regulation of association of proteasome components
80775	TMEM177	???
89870	TRIM15	???
91663	MYADM	??? Regulates the connection between the plasma membrane and the cortical cytoskeleton and so can control the endothelial inflammatory response
*114971*	*PTPMT1*	*Is an essential intermediate in the biosynthetic pathway of cardiolipin, a mitochondrial-specific phospholipid regulating the membrane integrity and activities of the organelle*
124583	CANT1	Functions as a calcium-dependent nucleotidase
127687	C1orf122	???
135932	TMEM139	???
140465	MYL6B	Regulatory light chain of myosin
140606	SELM	May function as a thiol-disulfide-oxidoreductase that participates in disulfide bond formation
147007	TMEM199	???
151613	TTC14	??? RNA binding
155066	ATP6V0E2	Play an important role in processes such as receptor-mediated endocytosis, protein degradation, and coupled transport
196383	RILPL2	Involved in cell shape and neuronal morphogenesis, positively regulating the establishment and maintenance of dendritic spines
252839	TMEM9	May be involved in intracellular transport
253461	ZBTB38	May be involved in the differentiation and/or survival of late postmitotic neurons
375757	C9orf119	Required for double-strand break repair via homologous recombination
389203	C4orf52	???
100128750	LOC100128750	???
100505687	LOC100505687	???

The description of the functionality of the gene has been taken from GeneCards; genes involved in mitochondrial processes according to the MITOP2 database are in italic

To enable biological interpretation, an overrepresentation analysis was performed on this set of variable genes, using the overrepresentation module of ConsensusPathDB (Kamburov et al. 2013). A background list consisting of all genes passing the selection as described under ‘transcriptomic data analysis’ was used in this analysis.

Subsequently, the correlation score matrices (see top left corner supplementary Figure 1) created for each of the top 1 % highly variable genes and all metabolites were used to find gene expression profiles that match metabolite profiles on an individual level. To do so, the interdonor correlations for all highly variable genes were correlated with the interdonor correlations from all metabolites (Pearson). A cutoff of >0.7 was used to define genes of which the difference in expression levels mimicked the difference in the metabolite levels between donors. The results of this analysis are summarized in supplementary Table 3.

To define genes related to mitochondrial processes, the top 1 % variable genes were compared to the mitochondrial reference gene set from MITOP2. This is a database which provides a list of human mitochondrial proteins linked to their gene names found through computational prediction of signaling sequences, but also includes results from proteome mapping, mutant screening, expression profiling, protein–protein interaction, and cellular sub-localization studies (Elstner et al. 2009). The MiMI plugin for Cytoscape was used to generate a network for all mitochondrial-related genes based on the nearest neighbor analysis (Fig. 3; Gao et al. 2009). Only the nearest neighbors shared by more than one of the mitochondrial-related genes were taken into consideration.

## Results

### Transcriptomics

Just over 10,000 genes were screened for interindividual variation in their responses toward APAP exposure by correlating their expression over dose. Standard deviations of these correlation scores showed a normal distribution. To assure that only the most variable genes were used for further analyses, and a short list was created of the top 1 % most variable genes (see Table 1, *n* = 99, SD > 2.52).

**Fig. 1:**
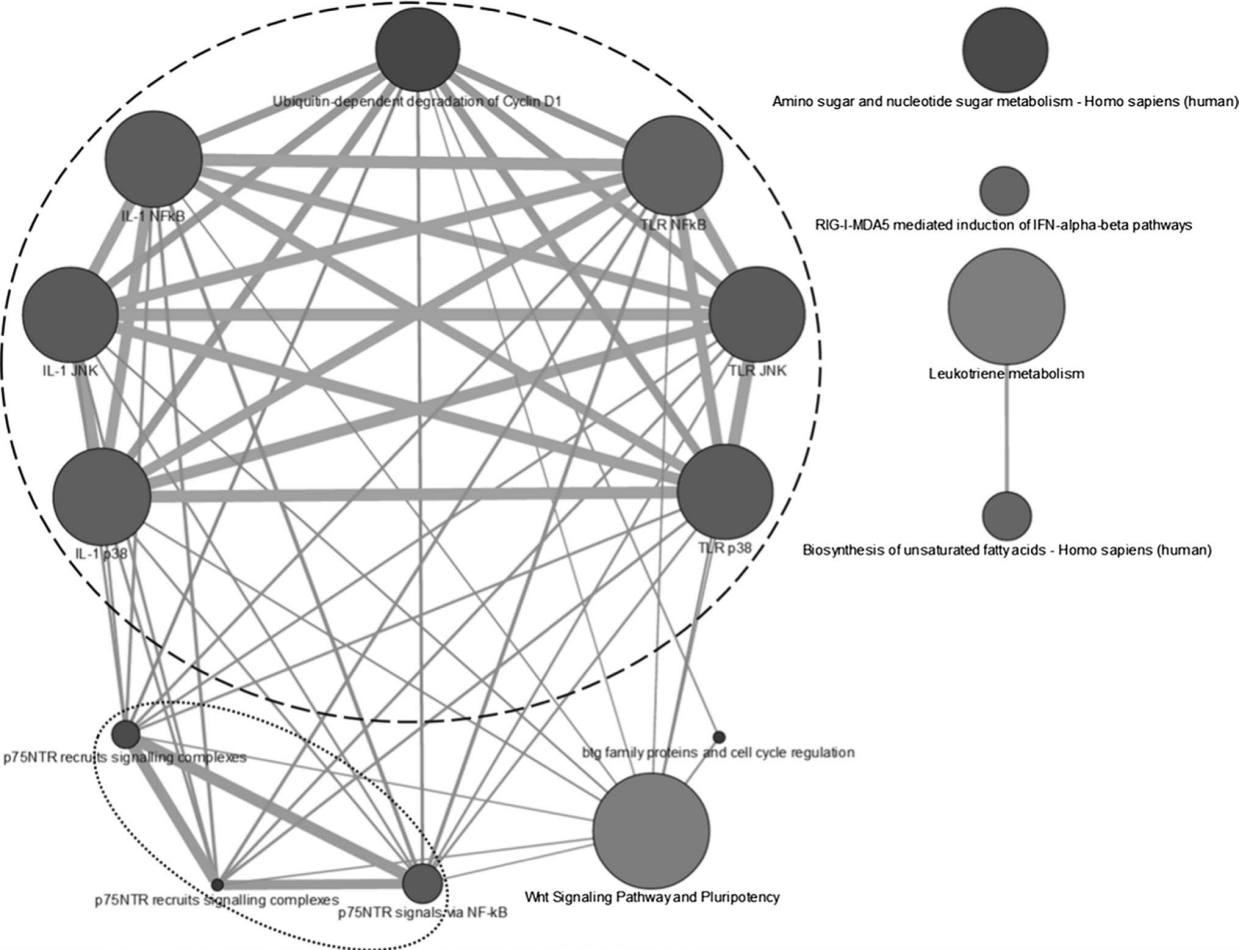
Network of the top 1 % most variable genes between donors after APAP exposure. The network was created based on a gene set overrepresentation analysis (ConsensusPathDB). Each node represents a biological pathway, the size of the node represents the amount of genes included in the pathway (*bigger diameter* = larger # genes), the *color* of the node represents its significance (*darker gray* = lower *p* value), and the *thickness* of the edge represents the amount of overlap between the connected nodes (*thicker line* = higher # overlapping genes)

To define the functionality of the variable genes, an overrepresentation analysis was performed, i.e., a network consisting of the biological pathways containing the genes with the highest variability between donors in response to APAP exposure was defined (see Fig. 1). This overrepresentation network could be broken down into several parts; one large component appeared constructed of:

- A large cluster with toll-like receptors (TLR), c-Jun N-terminal kinases (JNK), nuclear factor (NF)-κB, interleukin (IL)-1, p38, and cyclin D1-related pathways (encircled with striped line).
- A smaller cluster around p75 neurotrophin receptor (p75 NTR) (encircled with dotted line).
- Two more components, involved in ‘Wnt-signaling pathway and pluripotency’ and ‘BTG (B cell translocation gene) family proteins and cell cycle regulation.’

Additional parts consisted of components separated from the large cluster on ‘leukotriene metabolism,’ ‘biosynthesis of unsaturated fatty acids,’ ‘amino sugar and nucleotide sugar metabolism,’ ‘retinoic acid-inducible gene 1 (RIG-I), and melanoma differentiation-associated protein 5 (MDA5)-mediated induction of interferon (IFN)-alpha–beta.’

**Fig. 2:**
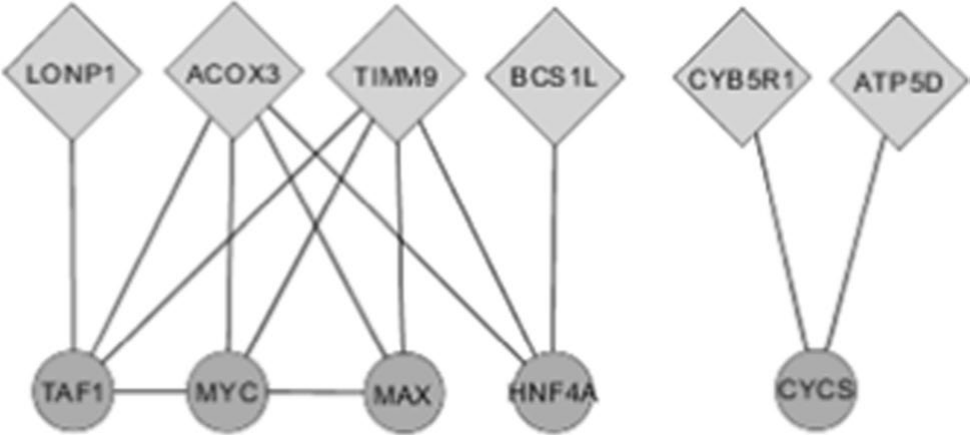
Network of highly variable mitochondrial-related genes. Nearest neighbor analysis was performed on all mitochondrial-related genes from the top 1 % highly variable gene list. Only the nearest neighbors shared by more than one of the variable genes were taken into account. *Square* nodes represent input genes, and *round* nodes represent the shared nearest neighbors

A further network based on mitochondrial-related genes form the top 1 % highly variable genes was created using next neighbor analysis in Cytoscape Fig. 2. This figure shows that transcription factors are the main binding element in the response of mitochondrial-related genes, showing high interindividual variation in gene expression response after APAP exposure.

### Metabolomics

**Fig. 3:**
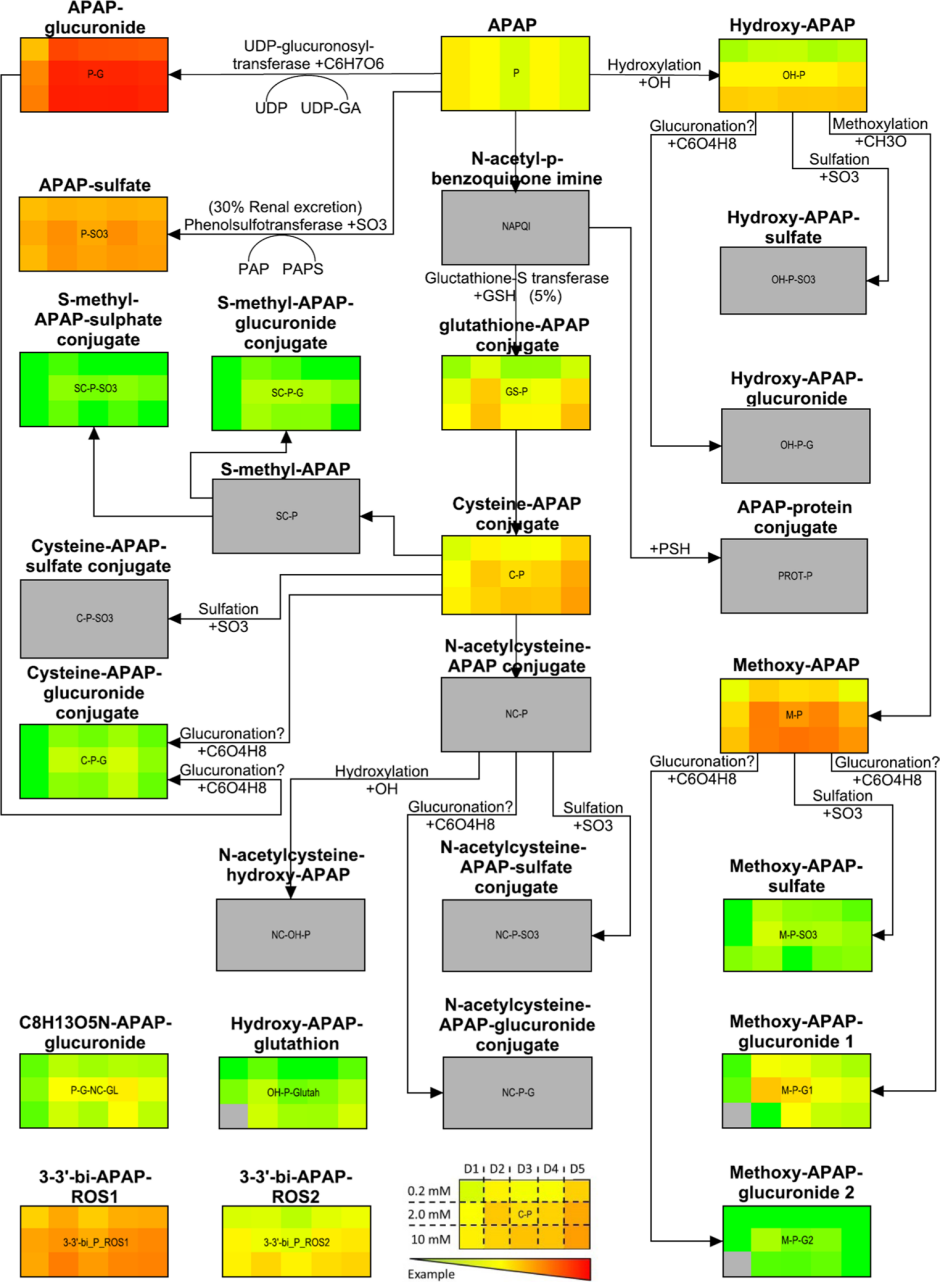
Schematic visualization of APAP metabolic pathway. The log-transformed metabolite levels for each donor on at each dose corrected for 0 mM are visualized. *Gray boxes* not measured/detected. Increase in a metabolite is pictured from *green* (no increase, equals a numerical value of 0 on a log scale) to *yellow*, *orange*, and *red* (high increase, maximum value = 5 on a log scale). Figure adapted from Jetten et al. (2012) (color figure online)

A broad spectrum of metabolites was measured in the medium, as shown in Supplementary Table 3 and Figure 3. In general, the variation between individuals was lower with respect to metabolite levels when compared to the variation in gene expression levels. To define how the variability between donors in gene expression is related to the variability in metabolite level in these same donors, a Pearson-based correlation analysis between the top 1 % variable genes and all metabolites was performed (cutoff *R*_2_ > 0.7). Out of the 99 most variable genes, 91 could be linked to the variation in metabolites on an individual level, meaning that these 91 genes can at least partially explain the interindividual variation observed in metabolites. In particular, hydroxy-APAP, methoxy-APAP, and the tentatively identified metabolite C_8_H_13_O_5_N-APAP-glucuronide showed strong correlations with genes on an individual level (*n* = 36, 36, and 51 correlating genes, respectively). Interestingly, C_8_H_13_O_5_N-APAP-glucuronide has previously been reported by Jetten et al. (2012) as a novel APAP metabolite, which could be detected in the in vivo human situation after low-dose APAP exposure. This metabolite could thus be confirmed in the current study in an in vitro human situation consisting of primary human hepatocytes. Furthermore, a mass tentatively assigned to 3,3′-biacetaminophen (not detected previously) has also been found. 3,3′-biacetaminophen has been suggested to result from NAPQI reacting with APAP and is considered a reactive oxygen species (ROS) product (Chen et al. 2008a).

## Discussion

The aim of this study is to evaluate the interindividual differences in gene expression changes and APAP metabolite formation in primary human hepatocytes of several donors (*n* = 5) exposed to a non-toxic to toxic APAP dose range.

Interindividual variation in gene expression is a very common phenomenon; therefore, we have focused on the gene expression changes that are most different between individuals in response to APAP exposure. To do so, we have created a short list consisting of the top 1 % most different genes based on correlation analysis (*n* = 99, see Table 1). Expression levels of many genes/metabolites including, but not limited to cytochrome P450 enzymes, glucuronosyltransferases, sulfotransferases, and glutathione S-transferases have been shown to influence the biotransformation processes of APAP (Zhao and Pickering 2011). However, studies in general link baseline expression levels of these genes to APAP metabolism parameters, while in the current study we focus on response parameters after APAP exposure in order to explain interindividual variability.

To define the biological functionality of the genes with the highest variability between individuals (top 1 % list), a network of pathways found by gene set overrepresentation analysis on this list was created (Fig. 1). This network shows a large cluster with TLRs, JNK, NF-κB, IL-1, p38, and cyclin D1-related pathways (encircled with striped line). TLR, JNK, and NF-κB pathways are all key regulators in the production of cytokines associated with inflammatory responses and the early stages of the development of hepatocarcinogenesis (Maeda 2010). Furthermore, all of the above-mentioned pathways have been associated with the process of liver regeneration (Iimuro and Fujimoto 2010). Hepatocytes rarely undergo proliferation in the liver under normal circumstances. However, proliferation can be triggered in response to loss of liver mass for instance induced by liver resection but also due to toxin-induced hepatocyte trauma like is the case with APAP. In both in vitro and in vivo studies, APAP has been shown to induce a persistent activation of JNK adding to hepatocellular necrosis (Gunawan et al. 2006). Cross talk between JNK and NF-κB has been proposed as a mechanism through which JNK mediates cell death. Matsumura et al. (2003) showed that inhibition of NF-κB in the liver by APAP leads to amplification of JNK and as such shifts the balance from cell survival toward cell death. IL-1 stimulates both the JNK and NF-κB pathways, which increases cell signaling and interferes with the cell cycle (Matsuzawa et al. 2005; Sanz-Garcia et al. 2013). TLR plays a role in the expression of cytokines and hepatomitogens; in response to TRL activation, p38 is triggered, leading to cytokine- and stress-induced apoptosis (Matsuzawa et al. 2005; Sanz-Garcia et al. 2013). Finally, it is indicated that that NF-κB activation operates on cell growth through cyclin D1 expression (Guttridge et al. 1999; Maeda 2010). In acetaminophen-induced liver injury, sustained JNK activation, through NF-κB, is essential in inducing apoptosis (Hanawa et al. 2008). Furthermore, it is suggested that NAPQI-induced damage on its own is not enough to cause hepatocyte death after APAP dosing and that activation of signaling pathways involving JNK is necessary to lead to cell death (Hanawa et al. 2008; Kaplowitz 2005). The actual downstream targets of JNK that are involved in APAP-induced liver injury are still largely unknown; however, a role for mitochondrial proteins has recently been suggested (Zhou et al. 2008).

Interestingly, 10 % of the genes from the top 1 % highly variable gene list consist of mitochondrial-related genes (see Table 1; genes in Italic). The majority of these genes are involved in metabolism-related processes [CCBL2 (Yu et al. 2006), PTPMT1 (Shen et al. 2011), ACOX3 (Cui et al. 2010), ACOT7 (Fujita et al. 2011), and CYB5R1 (Chae et al. 2013)], while others are part of the respiratory chain complexes in the mitochondria [ATP5D (Sotgia et al. 2012) and BCS1L (Kotarsky et al. 2012)] or are involved in more structurally-related processes like protein synthesis [MRPS26 (Sotgia et al. 2012)] and mitochondrial matrix or membrane maintenance [LONP1 (Tian et al. 2011) and TIMM9 (Sotgia et al. 2012), respectively]. All but one (MRPS26) of the above-mentioned genes has been related to the toxic/necrotic effects of APAP in a study comparing the toxicity response to APAP in rats/mice with the response to APAP’s far less toxic stereo-isomer N-acetyl-m-aminophenol [AMAP; (Beyer et al. 2007)]. In addition, ATP5D, MRPS26, LONP1, ACOT7, and TIMM9 were also shown to be regulated in HepG2 cells exposed to a toxic (10 mM) APAP dose for 12–72 h (unpublished results). Drug-induced liver injury has often been linked to regulation of the mitochondrial stress responses, which include the formation of ROS products, alterations in lipid metabolism, electron transport, cofactor metabolism, and the activation of pathways important in determining cell survival or death (Beyer et al. 2007; Han et al. 2013).

The nearest neighbor analyses on all mitochondrial-related genes from the top 1 % highly variable gene list show that mainly transcription factors seem to be the binding element in the response of the mitochondrial-related genes (Fig. 2). The involvement of transcription factors has been suggested in the drug-induced stress response of mitochondria in hepatocytes (Han et al. 2013). This indicates that interindividual variation exists in the response of mitochondrial-related genes, possibly explaining part of the differences between humans in mitochondrial-related APAP-induced toxicity responses and consequential liver damage levels.

Furthermore, in the gene set overrepresentation network, another smaller network around p75NTR is present (Fig. 1, encircled with dotted line). P75 NTR is a cell membrane receptor protein that has been associated with tumor and metastasis suppression (Khwaja et al. 2004), similar to the cluster described above. Non-steroidal anti-inflammatory drugs (NSAIDs) are used to reduce inflammation and also act as analgesics by the inhibition of cyclooxygenase-2 (COX-2). However, high concentrations of some NSAIDs are able to reduce proliferation and induce apoptosis in cancer cells. Several molecular mechanisms have been proposed as possible mediators in the anticancerous effects of NSAIDs, including p75NTR. Although APAP is not considered to be a real NSAID, due to its limited anti-inflammatory effects, APAP does affect similar pathways and works as an analgesic through COX-2 inhibition which might explain why similar effects have been suggested for APAP (Bonnefont et al. 2007).

Two other components in the gene set overrepresentation network that are connected to the components described above are the ‘Wnt-signaling pathway and pluripotency’ and ‘BTG family proteins and cell cycle regulation’ (see Fig. 1). Both pathways can be related to APAP-induced toxicity-related effects. The stimulation of the Wnt pathway has been suggested to be beneficial after APAP-induced liver failure by stimulating liver regeneration (Apte et al. 2009). This corresponds with the previously mentioned regulation of liver regeneration by the genes involved in the cluster around TLRs, JNK, NF-κB, IL-1, p38, and cyclin D1-related pathways. The BGT gene family has been associated with APAP hepatotoxicity before (Beyer et al. 2007) and has a function in DNA-strand break repair (Choi et al. 2012) and in the regulation of reactive oxygen species generation in the mitochondria (Lim et al. 2012). Both APAP and NAPQI are known to covalently bind to DNA and cause DNA damage (Dybing et al. 1984; Rannug et al. 1995), which possibly explains why this pathway is triggered by APAP exposure. As already described above, mitochondrial stress is an intricate part of the cellular response to APAP-induced oxidative stress (Hanawa et al. 2008), which might also explain the response of the BTG-related pathway. These findings agree with the previously mentioned interindividual variation in mitochondrial genes.

In addition, several other pathways not connected to the main cluster of pathways are included in the network of the gene set overrepresentation analysis. These are ‘leukotriene metabolism’ together with ‘biosynthesis of unsaturated fatty acids,’ ‘amino sugar and nucleotide sugar metabolism’ and ‘RIG-I-MDA5-mediated induction of IFN-alpha–beta.’ The first set of pathways (leukotrienes and fatty acids) probably represents the variation in the normal, therapeutic mechanism of APAP. In humans, unsaturated fatty acids are bioactivated through enzymatic oxygenation to, among others, prostaglandins and leukotrienes which contribute to fever, pain, inflammation, and cancer development (Ricciotti and FitzGerald 2011). APAP interferes with these processes as such inhibiting symptoms. Concerning the sugar metabolism pathway, carbohydrate homeostasis is essential for normal liver function. It is well known that during APAP-induced liver failure, these processes are severely affected (Record et al. 1975). Finally, RIG-I-MDA5-mediated induction of IFN-alpha–beta is a process that has been linked to several liver pathologies like hepatitis A/B/C and hepatic steatosis (Kawai et al. 2005; Toyoda et al. 2013; Wei et al. 2010). Although no apparent link with APAP is available in the literature, it seems that this process is somehow linked to an APAP-induced stress response.

To determine how the variation in the above-mentioned genes can explain the interindividual variation in metabolism levels, a correlation analysis was performed between the top 1 % most variable genes and all metabolites. Glucuronidation and sulfation are the two major processes in APAP metabolism, resulting in APAP-glucuronide and APAP-sulfate, respectively, which are non-toxic APAP metabolites (see Fig. 3; Chen et al. 2008a). In addition, another less abundant route of APAP metabolism utilizes hydroxylation/methoxylation of APAP. Hydroxy-APAP and methoxy-APAP are oxidative metabolites formed during this route of APAP metabolism, and both these metabolites have been associated with the hepatotoxic effects of APAP (Chen et al. 2008a; Dahlin et al. 1984b; Wilson et al. 1982). The variation in both these metabolites could be explained by a large proportion of the genes from the top 1 % most variable genes (*n* = 36 for both metabolites). It thus seems that the largest interindividual variation in gene expression responses after APAP exposure can be linked to the formation of toxic APAP metabolite formation. Interestingly, hydroxy/methoxy-derived metabolites could also be detected in human in vivo low-dose APAP exposure (Jetten et al. 2012). The fact that these metabolites and their derivatives are detectable both in vivo and in vitro, even at APAP doses within the therapeutic range and that their variation in expression levels between individuals can be linked to the variation in gene expression of genes related to toxicity-related effects of APAP exposure, indicates a role as potential key elements in the molecular mechanism behind APAP toxicity. Additionally, another metabolite, C_8_H_13_O_5_N-APAP-glucuronide, also shows correlation with a large set of the top 1 % variable genes (*n* = 51). This metabolite was described as new in the low-dose in vivo APAP exposure study of Jetten et al. (2012). Since this metabolite is relatively unknown, further studies on its exact route of metabolism and toxic potency could lead to further insight into the molecular mechanism behind APAP toxicity for the same reasons as explained for hydroxyl/methoxy-APAP above.

In summary, the biological processes in which the genes with the highest variability in expression between individuals after APAP exposure are involved can be linked to APAP-toxicity-related processes like liver regeneration, inflammatory responses, and mitochondrial stress responses. Also, processes related to hepatocarcinogenesis, cell cycle, and drug efficacy show large interindividual variation after APAP exposure.

In addition, most of the genes with high variability between individuals after APAP exposure can be linked to variability in expression levels of metabolites (hydroxyl/methoxy-APAP and C8H13O5N-APAP-glucuronide). Possibly, these findings could help explain the differences seen in susceptibility to APAP toxicity in the in vivo situation. Furthermore, they might give an indication for where the factors causing variability in susceptibility toward APAP-induced liver failure could be found.

## Electronic supplementary material

Below is the link to the electronic supplementary material.

Supplementary Figure 1 **Representative correlation plot**. X-axis: APAP dose range, Y-axis: Log2 gene/metabolite expression level, lines represent responses over dose per donor, arrows represent performed Pearson correlations, Corr. Score table (top left) shows the sum of absolute correlation score per donor (= absolute sum of value of arrows per donor). Supplementary material 1 (PDF 202 kb)

Supplementary Table 1 **Donor demographics**. Supplementary material 2 (PDF 177 kb)

Supplementary Table 2 **Identified masses derived from UPLC-TOF/MS after 24-h exposure to APAP.** For each of the detected metabolites its full name, abbreviation, mass, retention, and composite molecular groups are shown. . Supplementary material 3 (PDF 201 kb)

Supplementary Table 3 **Correlation between interindividual variation in gene expression levels and metabolite expression levels.** Pearson correlation analysis between the donor-specific correlation scores of log-transformed expression-values of the top 1% highly variable genes and all metabolites. Correlation coefficients > 0.70 are in bold. Supplementary material 4 (PDF 300 kb)
