# Supplementary figures and images for: Interindividual variation in gene expression responses and metabolite formation in acetaminophen-exposed primary human hepatocytes
Source: Arch Toxicol. 2015 Jun 24;90:1103–15. doi: 10.1007/s00204-015-1545-2 (PMC4830893; doi:10.1007/s00204-015-1545-2)

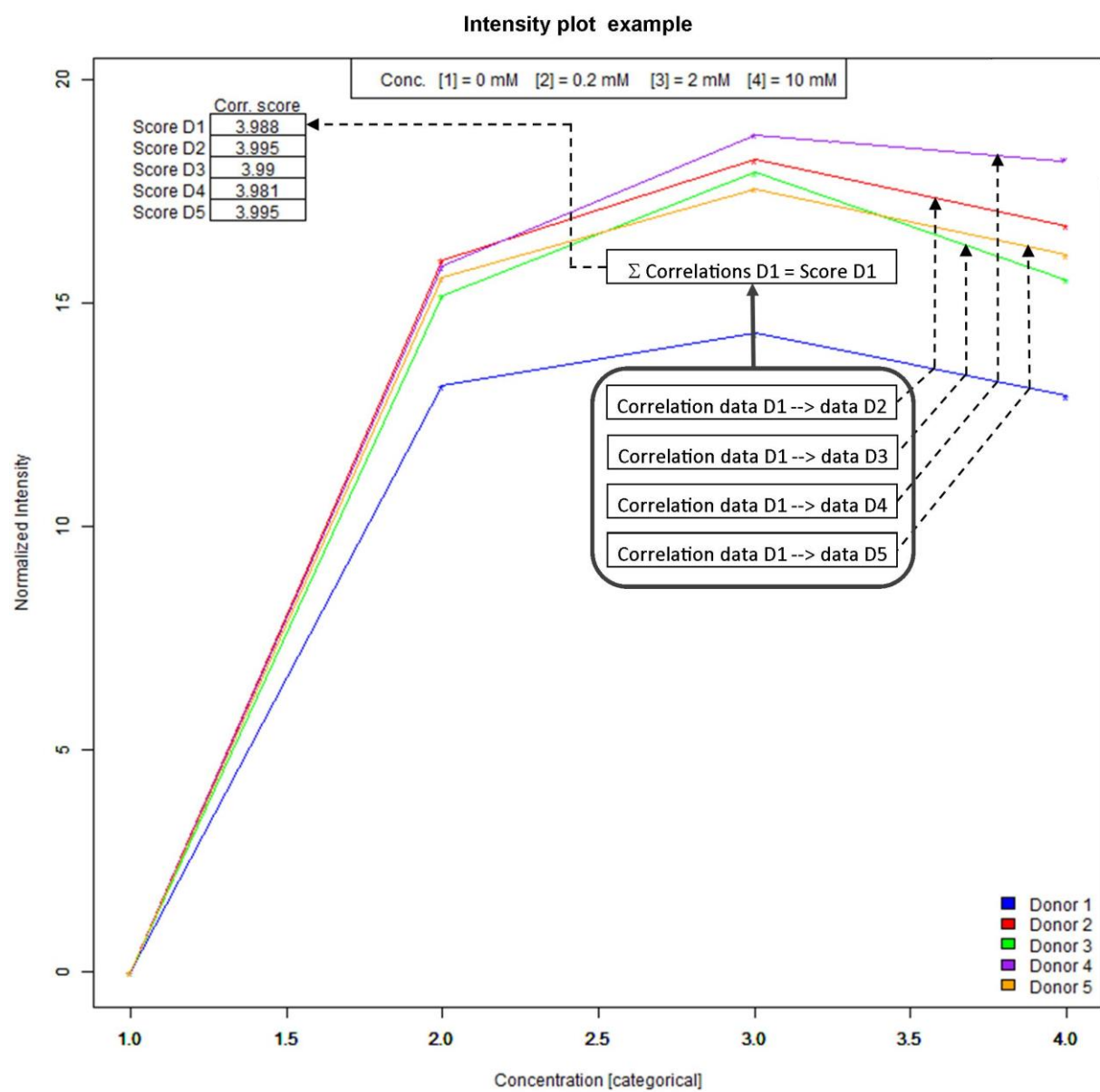

Supplementary Figure 1

Supplement: Supplementary file 1 — Representative correlation plot. X-axis: APAP dose range, Y-axis: Log2 gene/metabolite expression level, lines represent responses over dose per donor, arrows represent performed Pearson correlations, Corr. Score table (top left) shows the sum of absolute correlation score per donor (= absolute sum of value of arrows per donor). Supplementary material 1 (PDF 202 kb) [file 204_2015_1545_MOESM1_ESM.pdf]
