# Supplementary material for: Interindividual variation in gene expression responses and metabolite formation in acetaminophen-exposed primary human hepatocytes
Source: Arch Toxicol. 2015 Jun 24;90:1103–15. doi: 10.1007/s00204-015-1545-2 (PMC4830893; doi:10.1007/s00204-015-1545-2)
Supplement: Supplementary file 2 — Donor demographics. Supplementary material 2 (PDF 177 kb) [file 204_2015_1545_MOESM2_ESM.pdf]

Donor #	Sex	Race	Age (years)	Smoker	Alcohol use	Drug use	Cause of death
**1**	Female	Caucasian	36	No	Yes	No	Unknown
**2**	Female	Caucasian	63	Yes	No	No	Cerebrovascular accident
**3**	Female	Caucasian	57	No	Yes	No	Intra-cerebral hemorrhage
**4**	Female	Caucasian	57	No	No	No	Anoxia
**5**	Female	Caucasian	59	Yes	Yes	No	Cerebrovascular accident

Supplementary Table 1
