# Supplementary material for: Interindividual variation in gene expression responses and metabolite formation in acetaminophen-exposed primary human hepatocytes
Source: Arch Toxicol. 2015 Jun 24;90:1103–15. doi: 10.1007/s00204-015-1545-2 (PMC4830893; doi:10.1007/s00204-015-1545-2)
Supplement: Supplementary file 3 — Identified masses derived from UPLC-TOF/MS after 24-h exposure to APAP. For each of the detected metabolites its full name, abbreviation, mass, retention, and composite molecular groups are shown. . Supplementary material 3 (PDF 201 kb) [file 204_2015_1545_MOESM3_ESM.pdf]

Metabolite name	Metabolite abbreviation	Mass (Dalton)	Retention (minutes)	APAP	OH	O-CH3	Sulf.	Gluc.	Cys.	S-CH3	Glutath.	C8H13O5N
**APAP**	P	152.07	5.13	X								
**APAP-sulfate**	P-SO3	232.03	4.25	X			X					
**APAP-glucuronide**	P-G	328.10	3.63	X				X				
**Hydroxy-APAP**	OH-P	168.07	3.77	X	X							
**Hydroxy-APAP-glutathione**	OH-P-Glutath	473.13	6.62	X	X						X	
**Methoxy-APAP**	M-P	182.08	7.39	X	X	X						
**Methoxy-APAP-sulfate**	M-P-SO3	262.04	5.88	X	X	X	X					
**Methoxy-APAP-glucuronide1**	M-P-G1	358.11	5.42	X	X	X		X				
**Methoxy-APAP-glucuronide2**	M-P-G2	358.11	6.63	X	X	X		X				
**Cysteine-APAP conjugate**	C-P	271.07	4.27	X					X			
**Cysteine-APAP-glucuronide conjugate**	C-P-G	447.11	3.16	X				X	X			
**S-methyl-APAP-sulfate conjugate**	SC-P-SO3	278.01	8.93	X			X			X		
**S-methyl-APAP-glucuronide conjugate**	SC-P-G	374.09	7.80	X				X		X		
**$C_8H_{13}O_5N$-APAP-glucuronide**	P-G-NC-GL	531.18	5.04	X				X				X
**3-3'-bi-APAP-ROS1**	3-3'-bi-P-ROS1	301.12	11.22	X								
**3-3'-bi-APAP-ROS2**	3-3'-bi-P-ROS2	301.12	14.80	X								
**Glutathione-APAP conjugate**	GS-P	457.14	6.64	X							X	

Supplementary Table 2
