# Supplementary material for: Interindividual variation in gene expression responses and metabolite formation in acetaminophen-exposed primary human hepatocytes
Source: Arch Toxicol. 2015 Jun 24;90:1103–15. doi: 10.1007/s00204-015-1545-2 (PMC4830893; doi:10.1007/s00204-015-1545-2)
Supplement: Supplementary file 4 — Correlation between interindividual variation in gene expression levels and metabolite expression levels. Pearson correlation analysis between the donor-specific correlation scores of log-transformed expression-values of the top 1% highly variable genes and all metabolites. Correlation coefficients > 0.70 are in bold. Supplementary material 4 (PDF 300 kb) [file 204_2015_1545_MOESM4_ESM.pdf]

EntrezGeneID	Genename	C-P-G	OH-P	M-P	P-G	P-G-NC-GL	GS-P	P-SO3	SC-P-G	SC-P-SO3
**513**	**ATP5D**	0.06	0.00	0.27	0.08	**0.74**	0.00	0.18	0.07	0.07
**595**	**CCND1**	0.05	0.00	0.28	0.07	**0.74**	0.00	0.17	0.07	0.06
**617**	**BCS1L**	0.07	**0.89**	**0.73**	0.08	0.23	0.27	0.30	0.00	0.12
**988**	**CDC5L**	0.05	0.00	0.28	0.07	**0.74**	0.00	0.17	0.07	0.06
**1545**	**CYP1B1**	0.07	0.00	0.26	0.09	**0.72**	0.00	0.20	0.08	0.08
**1611**	**DAP**	0.06	0.00	0.28	0.08	**0.73**	0.00	0.18	0.07	0.07
**2669**	**GEM**	0.06	0.00	0.25	0.09	**0.73**	0.00	0.20	0.08	0.08
**2766**	**GMPR**	0.07	**0.89**	**0.74**	0.08	0.24	0.27	0.29	0.00	0.12
**3276**	**PRMT1**	0.06	0.00	0.27	0.09	**0.73**	0.00	0.19	0.08	0.08
**4302**	**MLLT6**	**1.00**	0.05	0.10	**0.99**	0.02	0.26	**0.72**	**0.93**	**0.93**
**4615**	**MYD88**	0.07	0.00	0.28	0.09	**0.74**	0.00	0.19	0.08	0.08
**5287**	**PIK3C2B**	0.06	0.00	0.25	0.08	**0.72**	0.00	0.19	0.07	0.07
**5300**	**PIN1**	0.06	**0.89**	**0.73**	0.08	0.23	0.28	0.30	0.00	0.12
**5523**	**PPP2R3A**	0.07	0.00	0.27	0.09	**0.74**	0.00	0.19	0.08	0.08
**5550**	**PREP**	0.06	**0.90**	**0.72**	0.08	0.22	0.29	0.29	0.00	0.12
**5584**	**PRKCI**	0.07	0.00	0.28	0.09	**0.74**	0.00	0.18	0.08	0.08
**5696**	**PSMB8**	0.07	0.00	0.25	0.09	**0.72**	0.00	0.20	0.08	0.08
**5699**	**PSMB10**	0.06	0.00	0.27	0.08	**0.74**	0.00	0.18	0.08	0.07
**6612**	**SUMO3**	0.06	0.00	0.27	0.08	**0.73**	0.00	0.18	0.07	0.07
**6942**	**TCF20**	0.07	**0.90**	**0.73**	0.09	0.22	0.28	0.31	0.00	0.13
**7264**	**TSTA3**	0.06	0.00	0.27	0.08	**0.74**	0.00	0.18	0.07	0.07
**7572**	**ZNF24**	0.06	0.00	0.26	0.08	**0.73**	0.00	0.19	0.07	0.07
**7965**	**AIMP2**	0.06	**0.89**	**0.73**	0.08	0.23	0.28	0.29	0.00	0.12
**8270**	**LAGE3**	0.07	**0.90**	**0.72**	0.09	0.21	0.28	0.31	0.00	0.13
**8310**	**ACOX3**	0.05	0.00	0.28	0.07	**0.74**	0.00	0.17	0.06	0.06
**8985**	**PLOD3**	0.06	0.00	0.27	0.08	**0.73**	0.00	0.18	0.07	0.07
**9343**	**EFTUD2**	0.06	0.01	0.27	0.08	**0.72**	0.00	0.18	0.08	0.07
**9361**	**LONP1**	0.06	0.00	0.26	0.09	**0.72**	0.00	0.19	0.07	0.08
**9470**	**EIF4E2**	0.07	0.00	0.28	0.09	**0.74**	0.00	0.19	0.08	0.08
**10093**	**ARPC4**	0.06	0.00	0.27	0.09	**0.73**	0.00	0.19	0.08	0.08
**10189**	**THOC4**	0.07	0.00	0.25	0.09	**0.72**	0.00	0.20	0.08	0.08
**10313**	**RTN3**	0.06	**0.89**	**0.72**	0.07	0.22	0.29	0.29	0.00	0.11
**10422**	**UBAC1**	0.06	**0.90**	**0.72**	0.08	0.21	0.28	0.31	0.00	0.12
**10807**	**SDCCAG3**	0.06	0.00	0.29	0.08	**0.75**	0.00	0.17	0.07	0.07
**10899**	**JTB**	0.05	**0.89**	**0.73**	0.07	0.24	0.29	0.27	0.00	0.10
**11068**	**CYB561D2**	0.06	**0.89**	**0.73**	0.07	0.24	0.28	0.28	0.00	0.11
**11142**	**PKIG**	0.06	**0.88**	**0.74**	0.08	0.24	0.27	0.29	0.00	0.12
**11252**	**PACSIN2**	0.05	0.00	0.27	0.07	**0.74**	0.00	0.17	0.07	0.06
**11332**	**ACOT7**	0.06	0.00	0.26	0.08	**0.73**	0.00	0.18	0.07	0.07
**23325**	**KIAA1033**	0.07	0.00	0.26	0.09	**0.72**	0.00	0.19	0.08	0.07
**23558**	**WBP2**	0.07	0.01	0.26	0.09	**0.72**	0.00	0.19	0.09	0.08
**26100**	**WIPI2**	0.07	**0.88**	**0.75**	0.08	0.24	0.26	0.30	0.00	0.12
**26505**	**CNNM3**	**1.00**	0.06	0.12	**0.99**	0.02	0.24	**0.75**	**0.91**	**0.93**
**27075**	**TSPAN13**	0.06	**0.89**	**0.72**	0.08	0.23	0.29	0.29	0.00	0.11
**29105**	**C16orf80**	0.06	**0.88**	**0.75**	0.08	0.25	0.27	0.29	0.00	0.11
**29927**	**SEC61A1**	0.05	0.00	0.28	0.07	**0.75**	0.00	0.17	0.07	0.06

**50640**	**PNPLA8**	0.06	0.00	0.27	0.08	**0.73**	0.00	0.18	0.07	0.07
**51094**	**ADIPOR1**	0.07	**0.87**	**0.77**	0.09	0.26	0.25	0.30	0.00	0.12
**51491**	**NOP16**	0.06	0.00	0.26	0.08	**0.72**	0.00	0.19	0.08	0.07
**51504**	**TRMT112**	0.07	**0.88**	**0.75**	0.08	0.25	0.26	0.30	0.00	0.12
**51523**	**CXXC5**	0.06	0.00	0.27	0.08	**0.73**	0.00	0.19	0.07	0.07
**51706**	**CYB5R1**	0.06	**0.90**	**0.72**	0.08	0.22	0.28	0.30	0.00	0.12
**54187**	**NANS**	0.07	**0.89**	**0.74**	0.08	0.24	0.27	0.30	0.00	0.12
**54606**	**DDX56**	0.07	0.00	0.26	0.09	**0.72**	0.00	0.19	0.08	0.08
**54941**	**RNF125**	0.07	0.29	0.06	0.03	0.26	**0.71**	0.00	0.12	0.00
**55062**	**WIPI1**	0.06	**0.90**	**0.73**	0.08	0.23	0.28	0.29	0.00	0.12
**55111**	**PLEKHJ1**	0.06	0.00	0.28	0.08	**0.75**	0.00	0.18	0.07	0.07
**55238**	**SLC38A7**	0.05	0.00	0.28	0.07	**0.75**	0.00	0.17	0.06	0.06
**55315**	**SLC29A3**	0.06	0.00	0.27	0.08	**0.73**	0.00	0.18	0.07	0.07
**55647**	**RAB20**	0.07	0.00	0.26	0.09	**0.72**	0.00	0.19	0.08	0.08
**55700**	**MAP7D1**	0.07	0.00	0.25	0.09	**0.70**	0.00	0.20	0.08	0.08
**55743**	**CHFR**	0.06	0.00	0.24	0.09	**0.72**	0.00	0.20	0.07	0.08
**55898**	**UNC45A**	0.07	**0.88**	**0.75**	0.09	0.25	0.26	0.30	0.00	0.12
**56005**	**C19orf10**	0.06	0.00	0.26	0.08	**0.73**	0.00	0.19	0.07	0.07
**56267**	**CCBL2**	0.06	0.00	0.27	0.08	**0.74**	0.00	0.18	0.07	0.07
**56910**	**STARD7**	0.07	**0.90**	**0.73**	0.09	0.23	0.28	0.30	0.00	0.13
**57409**	**MIF4GD**	0.06	**0.89**	**0.74**	0.08	0.24	0.28	0.28	0.00	0.12
**64754**	**SMYD3**	0.06	**0.89**	**0.73**	0.08	0.23	0.28	0.29	0.00	0.11
**64787**	**EPS8L2**	0.07	0.00	0.24	0.09	**0.71**	0.00	0.21	0.08	0.08
**64949**	**MRPS26**	0.06	**0.89**	**0.73**	0.08	0.23	0.28	0.29	0.00	0.12
**66036**	**MTMR9**	0.07	0.00	0.26	0.09	**0.73**	0.00	0.19	0.08	0.08
**80227**	**PAAF1**	0.06	**0.89**	**0.74**	0.08	0.24	0.27	0.29	0.00	0.12
**80775**	**TMEM177**	0.06	**0.89**	**0.73**	0.08	0.23	0.28	0.29	0.00	0.12
**89870**	**TRIM15**	0.06	0.00	0.26	0.08	**0.73**	0.00	0.19	0.07	0.07
**91663**	**MYADM**	0.06	0.00	0.27	0.08	**0.72**	0.00	0.19	0.08	0.07
**114971**	**PTPMT1**	0.06	**0.89**	**0.73**	0.08	0.24	0.29	0.28	0.00	0.11
**124583**	**CANT1**	0.06	0.00	0.27	0.09	**0.73**	0.00	0.19	0.08	0.08
**127687**	**C1orf122**	0.06	0.00	0.27	0.08	**0.74**	0.00	0.18	0.07	0.07
**135932**	**TMEM139**	0.06	0.00	0.26	0.09	**0.72**	0.00	0.19	0.08	0.07
**140465**	**MYL6B**	0.07	0.00	0.26	0.09	**0.72**	0.00	0.19	0.08	0.08
**140606**	**SELM**	0.06	**0.89**	**0.74**	0.08	0.24	0.27	0.29	0.00	0.12
**147007**	**TMEM199**	0.07	**0.88**	**0.74**	0.09	0.23	0.26	0.32	0.00	0.13
**151613**	**TTC14**	0.06	0.00	0.27	0.08	**0.72**	0.00	0.19	0.08	0.07
**155066**	**ATP6V0E2**	0.07	**0.90**	**0.72**	0.09	0.22	0.28	0.30	0.00	0.13
**196383**	**RILPL2**	0.07	**0.90**	**0.73**	0.09	0.24	0.27	0.29	0.00	0.13
**252839**	**TMEM9**	0.06	**0.89**	**0.73**	0.08	0.24	0.28	0.29	0.00	0.12
**375757**	**ZBTB38**	0.05	**0.89**	**0.73**	0.06	0.24	0.30	0.27	0.00	0.10
**389203**	**C9orf119**	0.06	**0.89**	**0.73**	0.07	0.24	0.29	0.28	0.00	0.11
**100128750**	**LOC100128750**	0.07	0.00	0.26	0.09	**0.71**	0.00	0.19	0.08	0.07
**100505687**	**LOC100505687**	0.06	**0.89**	**0.73**	0.08	0.23	0.28	0.29	0.00	0.11

Supplementary Table 3
